# Resistance of *Francisella Novicida* to Fosmidomycin Associated with Mutations in the Glycerol-3-Phosphate Transporter

**DOI:** 10.3389/fmicb.2012.00226

**Published:** 2012-08-09

**Authors:** Ryan S. Mackie, Elizabeth S. McKenney, Monique L. van Hoek

**Affiliations:** ^1^School of Systems Biology, George Mason UniversityManassas, VA, USA; ^2^Naval Surface Warfare Center – Dahlgren DivisionDahlgren, VA, USA; ^3^National Center for Biodefense and Infectious Diseases, George Mason UniversityManassas, VA, USA

**Keywords:** *Francisella*, fosmidomycin, resistance, glycerol-3-phosphate transporter

## Abstract

The methylerythritol phosphate (MEP) pathway is essential in most prokaryotes and some lower eukaryotes but absent from human cells, and is a validated target for antimicrobial drug development. The formation of MEP is catalyzed by 1-deoxy-d-xylulose 5-phosphate reductoisomerase (DXR). MEP pathway genes have been identified in many category A and B biothreat agents, including *Francisella tularensis*, which causes the zoonosis tularemia. Fosmidomycin (Fos) inhibits purified *Francisella* DXR. This compound also inhibits the growth of *F. tularensis* NIH B38, *F. novicida* and *F. tularensis* subsp. *holarctica* LVS bacteria. Related compounds such as FR900098 and the lipophilic prodrug of FR900098 (compound 1) have been developed to improve the bioavailability of these DXR inhibitors. In performing disk-inhibition assays with these compounds, we observed breakthrough colonies of *F. novicida* in the presence of Fos, suggesting spontaneous development of Fos resistance (Fos^R^). Fos^R^ bacteria had decreased sensitivity to both Fos and FR900098. The two most likely targets for the development of mutants would be the DXR enzyme itself or the glycerol-3-phosphate transporter (GlpT) that allows entry of Fos into the bacteria. Sensitivity of Fos^R^ *F. novicida* bacteria to compound 1 was not abated suggesting that spontaneous resistance is not due to mutation of DXR. We thus predicted that the *glpT* transporter may be mutated leading to this resistant phenotype. Supporting this, transposon insertion mutants at the *glpT* locus were also found to be resistant to Fos. DNA sequencing of four different spontaneous Fos^R^ colonies demonstrated a variety of deletions in the *glpT* coding region. The overall frequency of Fos^R^ mutations in *F. novicida* was determined to be 6.3 × 10^−8^. Thus we conclude that one mechanism of resistance of *F. novicida* to Fos is caused by mutations in GlpT. This is the first description of spontaneous mutations in *Francisella* leading to Fos^R^.

## Introduction

*Francisella tularensis* is the etiologic agent of tularemia, a zoonotic disease that occurs in much of the northern hemisphere including North America. Its potential use as a biological weapon has given this zoonotic organism much attention (Foley and Nieto, 2010). Ulceroglandular forms of the disease can be contracted by humans following interaction with small rodents and lagomorphs, as well as through mechanical and vector transmission by biting arthropods (Akimana and Kwaik, 2011; Potz-Biedermann et al., 2011). In addition, pneumonic cases of tularemia are occasionally seen in humans following inhalation of aerosols containing the bacteria (Matyas et al., 2007; Ojeda et al., 2008). The outcome of these various forms of disease is determined by the availability of prompt treatment with a variety of antibiotics (Ikaheimo et al., 2000; Greenfield and Bronze, 2004). These small, Gram-negative coccobacilli are susceptible to treatment with antimicrobial agents, including streptomycin, gentamicin, doxycycline, quinolones, and chloramphenicol (Scheel et al., 1992; Ikaheimo et al., 2000; Johansson et al., 2002). *F. tularensis* subsp. *holarctica*, which is found in the northern parts of Europe and Asia, consists of Biovars I and II that are susceptible or resistant to erythromycin, respectively (Kudelina and Olsufiev, 1980; Gestin et al., 2010). Resistance to penicillin-class β-lactams has been routinely demonstrated from naturally occurring isolates of *Francisella* due to the expression of β-lactamase (Bina et al., 2006).

The methylerythritol phosphate (MEP) pathway is essential in most prokaryotes and some lower eukaryotes but absent from human cells, and is a validated target for antimicrobial drug development (Wiemer et al., 2010). The formation of MEP is catalyzed by 1-deoxy-d-xylulose 5-phosphate reductoisomerase (DXR). MEP pathway genes have been identified in many category A and B biothreat agents, including *F. tularensis*, *Bacillus anthracis*, and *Yersinia pestis*. There are two well-known inhibitors of DXR, an enzyme responsible for the formation of MEP used for the down-stream synthesis of the isoprenoids (Jawaid et al., 2009). One is fosmidomycin (Fos, 3-[formyl(hydroxy) amino] propylphosphonic acid), currently in clinical trials for treatment of malaria (Wiesner et al., 2003), and the second is FR900098 (3-[acetyl(hydroxy)amino] propylphosphonic acid). We have previously demonstrated that Fos inhibits *Francisella* growth and can target purified *Francisella* DXR enzyme (Jawaid et al., 2009). Because the MEP pathway is found in most prokaryotes and lower eukaryotes, but not in humans, and is essential for survival of these organisms, it has been described as a validated target for the development of new antimicrobial therapies (Rodriguez-Concepcion, 2004; Singh et al., 2007; Davey et al., 2011).

**Figure 1:**
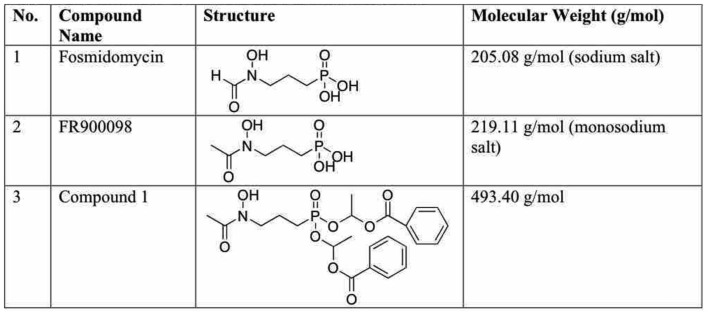
**Structure of inhibitors used in this study**. (1) Fosmidomycin (Fos, 3-[formyl(hydroxy) amino] propylphosphonic acid). (2) FR900098 (3-[acetyl(hydroxy)amino] propylphosphonic acid). (3) Compound 1: acyloxyalkyl ester prodrug derivative of FR900098 (Ortmann et al., 2003).

The glycerol-3-phosphate transporter (GlpT) system has been well characterized in *E. coli*, *B. subtilis*, and *Pseudomonas aeruginosa* (Nilsson et al., 1994; Lemieux et al., 2005; Castaneda-Garcia et al., 2009). GlpT is a member of the Major Facilitator Superfamily (MFS) that functions as an antiporter moving glycerol-3-phosphate into the cell, and exporting intracellular phosphate. In *E. coli*, the structure of the transporter consists of 12 membrane-spanning sections in alpha-helix form, joined together on the cytoplasmic side by an extended loop structure (Lemieux et al., 2005). A similar arrangement is predicted for *Francisella* GlpT. We have previously shown that the *Francisella* genome contains *glpT*, and that Fos effectiveness is mediated by the action of this transporter (McKenney et al., in press). The polar nature of Fos requires it be transported across the bacterial cell membrane via a transporter such as GlpT. Organisms that have DXR but lack such transporters, such as *Mycobacterium tuberculosis* and *Brucella abortus*, cannot transport Fos, and thus are functionally resistant (Brown and Parish, 2008; Sangari et al., 2010). However, when *E. coli* GlpT is expressed in *B. abortus*, Fos is transported into the bacterial cell, interacts with the DXR-like enzyme, and leads to decreased bacterial growth (Sangari et al., 2010). A series of acyloxyalkyl ester prodrug derivatives of FR900098, including compound 1 (Figure 1), have been generated which are cleaved to FR900098 by the bacterial esterases. They are more lipophilic in nature, demonstrate improved *in vitro* activity against *M. tuberculosis* (Uh et al., 2011), and improved *in vivo* activity (Ortmann et al., 2003) against malaria. We have demonstrated that the lipophilic prodrug of FR900098, compound 1, is able to bypass the Fos transporter and exert antimicrobial effects regardless of mutations in *glpT* that lead to Fos resistance (Fos^R^; McKenney et al., in press).

*Francisella* species are not known to be highly multi-drug resistant, or to rapidly develop resistance. *Francisella* are susceptible to many common antibiotics, except penicillins (Ikaheimo et al., 2000; Urich and Petersen, 2008). *Francisella* has two TolC-like proteins, TolC and the highly related FltC (Gil et al., 2006); mutations in these genes increase the sensitivity of *F. tularensis* LVS to various antibiotics, suggesting at least some role for drug efflux in the baseline sensitivity of *Francisella* to antibiotics. The sensitivity to macrolides varies between *Francisella* strains (Ahmad et al., 2010). *F. tularensis* LVS has a point mutation in Domain V of the 23S rRNA, rendering it more resistant to erythromycin than *F. novicida* or *F. tularensis* Schu S4 (Biswas et al., 2008). In the North American Type A *Francisella* strains, erythromycin MICs range from 0.5 to 4 μg/ml, while *F. tularensis* LVS has an MIC > 256 μg/ml (Marinov et al., 2009). Spontaneous antibiotic resistance to spectinomycin has been reported in *Francisella* at a low frequency of incidence (Kormilitsyna and Marakusha, 1983).

Here we report the spontaneous development of a Fos-resistant phenotype in *Francisella*. Spontaneous Fos^R^ has not been previously reported for this organism, and it is of concern to have a biothreat agent spontaneously acquire resistance to new classes of antibiotics. Thus we sought to understand the mechanism of Fos^R^ in *Francisella*.

## Materials and Methods

### Strains and growth of bacteria

*Francisella tularensis* subsp. *novicida* Utah 112 (*F. novicida*; BEI Resources # NR13) was grown in trypticase soy broth with 0.1% cysteine HCl (TSB-C), on TSB-C agar plates, or Chocolate II Agar plates (CHAB – BD Biosciences) as noted for each assay. *F. tularensis* subsp. *tularensis* NIH B38 (ATCC 6223; BEI Resources # NR50, deposited as the type strain for *F. tularensis tularensis*) was grown on TSB-C agar plates at 37°C with 5% CO_2_ for 48 h, harvested by scraping into 20% glycerol, and stored at −80°C. *Francisella* on plates was grown at 37°C in an atmosphere of 5% CO_2_. Minimum inhibitory concentration (MIC) assays were performed in cation-adjusted Mueller–Hinton broth (MHB) according to the guidelines published by the Clinical and Laboratory Standards Institute; M11-A7.

**Figure A1:**
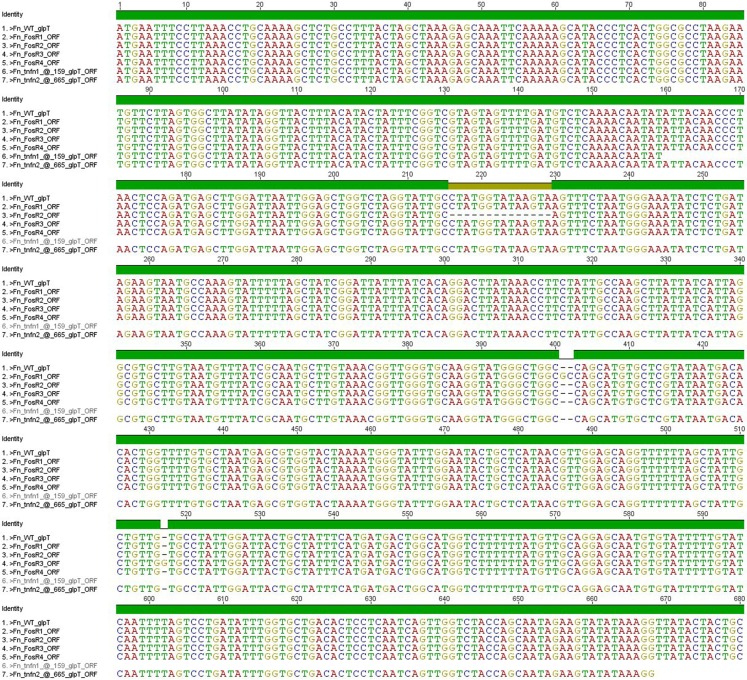

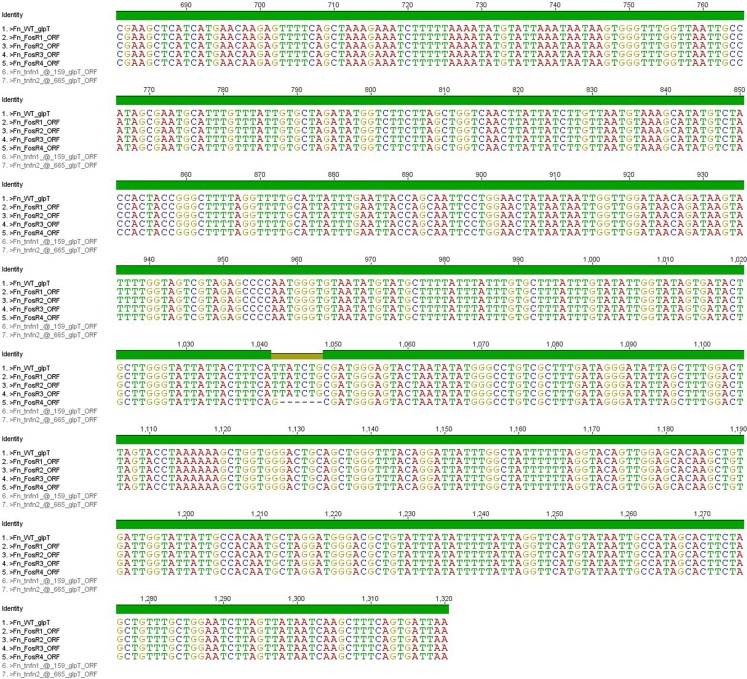
**Full DNA sequence data for Fos^R^ mutants vs. WT *F. novicida***. Detailed sequence results demonstrating the deletion and addition of nucleotides in a variety of locations in the full length open reading frame compared to the WT *F. novicida glpT* gene.

Transposon insertion mutants in the *glpT* locus [FTN_0636; *F. novicida* tnfn1_pw060323p08q150 BEI Catalog #NR-5683 and *F. novicida* tnfn1_pw060418p01q161 BEI Catalog #NR-6558, both Type T20 (IS*Fn*2/FRT)] (Gallagher et al., 2007) were obtained through the NIH Biodefense and Emerging Infectious Disease Research Repository, NIAID, NIH: *F. novicida*, “Two-Allele” Transposon Mutant Library. *F. novicida* U112 *glpT-1* and *glpT-2* (data not shown) transposon insertion mutants were grown as above, but in the presence of kanamycin (10 μg/ml) to select for the mutants. Sequences for the transposon mutants are shown in Figure A1 in Appendix (Gallagher et al., 2007).

### Preparation of stock solutions of Fos, FR900098, and compound 1

All inhibitor stocks were made at high starting concentration between 10 and 20 mg/ml. Fos sodium salt (Invitrogen #F-23103) and FR900098 monosodium salt (Sigma–Aldrich # F8307) were obtained as dry powders and were dissolved in water. Compound 1 (kind gift from C. Dowd) was dissolved in 100% DMSO to the final concentrations indicated.

### Disk-inhibition assay

Following the Kirby–Bauer disk diffusion assay protocols (Bauer et al., 1966), inhibitor-saturated disks were placed on a lawn of bacteria to display a zone of growth inhibition around the disk indicating susceptibility. A concentration of an overnight culture of bacteria equal to a 0.5 McFarland standard was spread on Chocolate II Agar plate (BD Biosciences) using a sterile cotton swab. Inhibitor disks were made by absorbing 20 μl of each inhibitor stock (10 mg/ml) to a sterile, thick Whatman filter paper 6-mm disk, so that each disk contained 200 μg. Three disks of the same inhibitor were added to each plate, except for NIHB38, for which only one disk was used per plate. For NIHB38 experiments, both 200 and 100 μg per disk were tested. The plates were wrapped in tin foil to protect the inhibitors from light and placed in the 37°C incubator for 48 h. The zone of inhibition was determined by measuring the diameter of the zone of no growth, including the 6-mm disk. Each zone was measured three times at different points of the zone (*n* = 9). A 6-mm zone of inhibition indicates no inhibition.

### MIC assays for Fos and lipophilic analogs against wild-type *F. novicida* U112 and transposon insertion mutants of *GlpT*

In accordance with CLSI standards, 150 μl cation-adjusted MHB was inoculated with 1.5 × 10^5^ CFU of *F. novicida*. For MIC assays against wild-type *F. novicida*, each well of column 1 was amended with 150 μl of Fos or compound 1 to achieve a final concentration of 300 and 200 μg/ml, respectively. Subsequent MIC assays had a starting concentration of 200 μg/ml regardless of the compound tested. Twofold serial dilutions of column 1 were performed to fill columns 2–11 of a 96-well plate. For compound 1, DMSO vehicle control wells were included in side-by-side assays. Column 12 received no drug and acts as the positive growth control for the assay. Absorbances (*A*_600_) were read for each well before incubation to record background. Plates were incubated at 37°C plus 5% CO_2_, *A*_600_ were determined at 24 and 48 h, and IC_50_ and MIC were determined.

### Genomic DNA preparation, PCR amplification, and sequencing

Overnight cultures were centrifuged to form bacterial pellets and supernatants were removed. Genomic DNA was prepared using the Wizard Genomic DNA kit (Promega, Madison, WI, USA). Briefly, pellets were re-suspended in 400 μl of nuclei lysis solution and incubated at 80°C for 15 min to lyse bacteria. Total DNA concentrations were analyzed using the Nanodrop spectrophotometer (Thermo Scientific, Wilmington, DE, USA) and adjusted to 40 ng/μl. Primers used for PCR confirmation that colonies were indeed *F. novicida* were designed to amplify 16S rRNAs (16SrRNAF: CTGTCGTCAGCTCGTGTTGT; 16SrRNA R: CGTAAGGGCCATGATGACTT; Forsman et al., 1994). Primers used to amplify the entire ORF of the *glpT* gene (*glpT*F: AACAGCGGTTTAGCTATTTTCAA; *glpT*R: TGCAATCTGAGCTGACTGAAG) were designed using Primer 3 through the National Center for Biotechnology Information (NCBI). Real-time PCR was performed on the Biorad MyIQ instrument with iQ SYBR Green Supermix in 50 μl reactions (Biorad, Hercules, CA, USA). Thermal cycler profile was set to 95°C for 3 min for initial denaturation and enzyme activation followed by 40 cycles of 95°C for 10 s and 55°C for 30 s. Sequencing primers were the same as the ORF amplification primers, but were synthesized and used by Functional Biosciences (Madison, WI, USA).

### Spontaneous mutation frequency for Fos^R^

A stock culture of *F. novicida* U112 was grown in TSB-C broth overnight at 37°C with continuous shaking at 165 rpm. Viable bacteria from overnight cultures were enumerated by serial dilution in sterile phosphate-buffered saline (PBS) and plated on TSB-C non-selective agar plates. TSB-C plates were incubated for >24 h, and colony forming units were counted to determine bacterial density of the culture. A diluted subculture was created to achieve a final concentration of 1 × 10^3^ CFU/ml. Parallel cultures prepared as 1 ml aliquots from the subculture (1 × 10^3^ CFU) were incubated overnight at 37°C with continuous shaking at 165 rpm (Rosche and Foster, 2000). The number of mutations that resulted in Fos^R^ was determined by plating 0.1 ml aliquots of the parallel cultures (*n* = 5) on TSB-C plates containing 122 μM (25 μg/ml) Fos. The total number of bacteria in the cultures was determined by serial dilution and plating on non-selective media (Rosche and Foster, 2000).

## Results

We have previously reported the *in vitro* antimicrobial activity of Fos and FR900098 (Figure 1) against *Francisella*, and the inhibition of purified *Francisella* DXR enzyme by these compounds (Jawaid et al., 2009; McKenney et al., in press). We have also demonstrated that a lipophilic prodrug of FR900098 (compound 1) is able to bypass the GlpT transporter and exert antimicrobial effects regardless of mutations in *glpT* that lead to Fos^R^, and that compound 1 is effective in an *in vivo* model of *F. novicida* infection (McKenney et al., in press).

### Wild-type *F. novicida*

**Figure 2:**
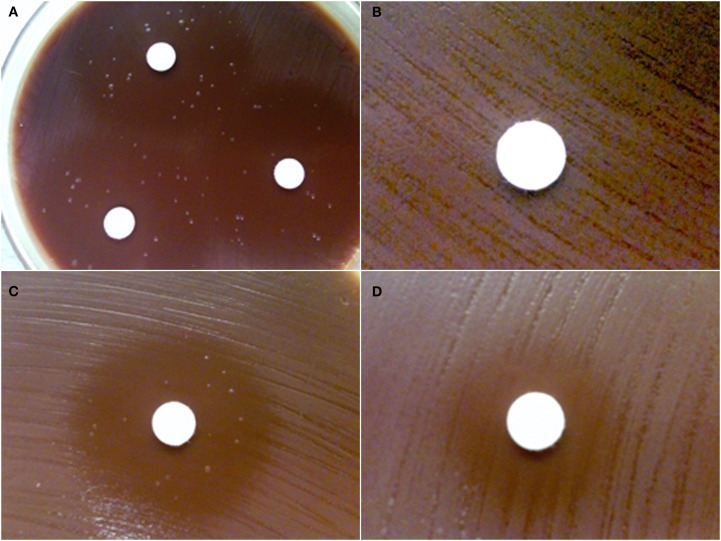
**Disk-inhibition assay**. Each disk contains 200 μg of antibiotic. **(A)** Wild-type *F. novicida* has a 37 ± 1-mm zone of inhibition around the disk containing fosmidomycin with multiple breakthrough colonies shown. **(B)** *GlpT-1* transposon mutant of *F. novicida* is completely resistant to fosmidomycin (zone of inhibition = 6 mm). **(C)** Wild-type *F. novicida* has a 29 ± 2-mm zone of inhibition around the disk containing FR900098. Breakthrough colonies can also be seen in the inhibition zone. **(D)** *GlpT-1* transposon mutant of *F. novicida* has intermediate resistance to FR900098 (zone of inhibition = 15 mm), but does not demonstrate any breakthrough colonies. This experiment was performed three times, with representative examples shown.

The Kirby–Bauer disk-inhibition assay was used to determine the susceptibility of wild-type *F. novicida* U112 to Fos and FR900098. The zone of inhibition around the disk containing 200 μg of Fos was 37 ± 1 mm (Figure 2A). The zone of inhibition around the disk containing 200 μg of FR900098 was 29 ± 2 mm (Figure 2C). For *F. tularensis* NIHB38, the type strain for Type A tularemia, both of these compounds lead to a large zone of inhibition. For a disk with 100 μg Fos, the zone of inhibition for *F. tularensis* NIHB38 was 72 ± 2 mm (data not shown), suggesting that *F. tularensis* Type A strains are sensitive to Fos. A zone of inhibition of 6 mm (the size of the disk) indicated no inhibition, which was observed for a water-only disk (data not shown).

### Spontaneous Fos^R^ mutants

While performing the Kirby–Bauer disk-inhibition assay with *F. novicida* against Fos and FR900098, breakthrough colonies were observed growing inside the zone of inhibition (Figure 2A – Fos, Figure 2C – FR900098). However, the zone of inhibition is still clearly defined. The colonies within the zone of inhibition are slightly different in their appearance than the wild-type *F. novicida* that is growing outside the zone of inhibition. The colonies are slightly larger and more mucoid, but are the same color as the wild-type colonies. These colonies (from Figures 2A,C) were confirmed to be *F. novicida* by real-time PCR using *F. novicida* specific 16S rRNA primers (Forsman et al., 1994; data not shown). Numerous breakthrough colonies were also observed for *F. tularensis* NIHB38 with both of these compounds, indicating that this “type strain” for *F. tularensis* Type A strains may also develop spontaneous resistance to Fos.

Based on our results in the disk-inhibition assay as well as our previous studies (McKenney et al., in press), we hypothesized that the two most likely bacterial targets for the development of mutants would be mutations of the DXR enzyme itself or mutations of GlpT that allow entry of Fos into the bacteria.

### MICs for wild-type and Fos^R^ mutants

**Figure 3:**
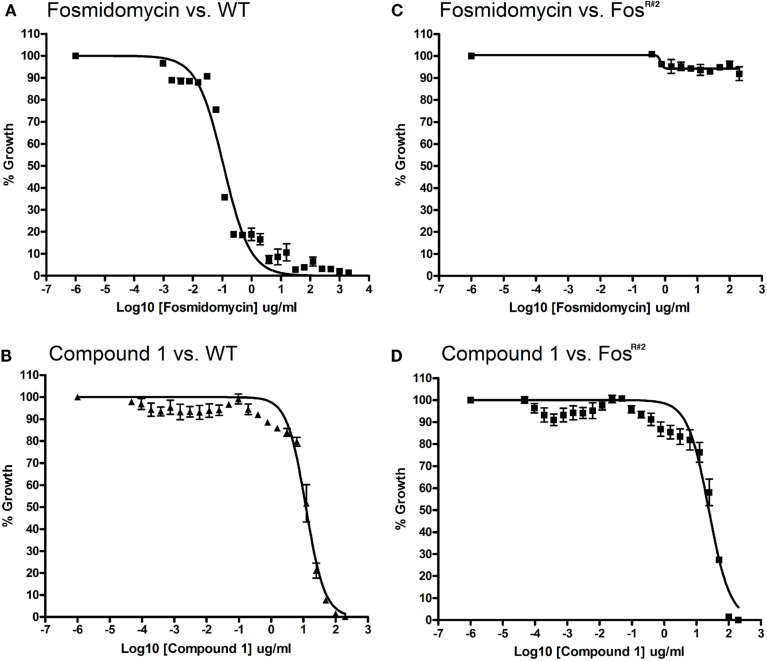
**Minimum inhibitory concentration assay for *F. novicida* and Fos^R#2^ mutant**. Wild-type *F. novicida* susceptibility to fosmidomycin **(A)** and compound 1 **(B)**. Wild-type MIC for fosmidomycin is 121 μM. [WT IC_50_ for fosmidomycin is 0.092 mg/l (95% CI = 0.079–0.107 mg/l)]. Wild-type MIC for compound 1 is 203 μm. Wild-type IC_50_ for compound 1 is 11.99 mg/l (95% CI = 9.84–14.62 mg/l, *R*^2^ = 0.972). Fos^R#2^ mutant resistance to fosmidomycin **(C)** Fos^R#2^ and susceptibility to compound 1 **(D)**. *F. novicida glpT* mutant MIC or IC_50_ for fosmidomycin could not be defined due to lack of inhibition (*R*^2^ value for fitness = 0.38). Fos^R#2^ mutant MIC for compound 1 is approximately 203 μM. The IC_50_ for compound 1 is 24.38 mg/l (95% CI = 18.75–31.70 mg/l, *R*^2^ = 0.944).

We found that Fos^R^ mutants are resistant to Fos but are sensitive to compound 1, the lipophilic prodrug of FR900098. The MIC for Fos against wild-type *F. novicida* is 121 μM (IC_50_ is 0.101 μg/ml with a 95% confidence interval (CI) = 0.079–0.130 mg/l; Figure 3A). The MIC for the lipophilic compound 1 against *F. novicida* is 100 μg/ml or 203 μM (IC_50_ is 11.99 μg/ml, 95% CI = 9.84–14.62 μg/ml; Figure 3B). A Fos-resistant colony (Fos^R#2^) isolated from our disk-inhibition assays was grown overnight as described, and the MICs and IC_50_s of Fos and compound 1 were determined. Concentrations of inhibitor used in this assay ranged from 0.4 to 200 μg/ml. Fos^R#2^-resistant bacteria grew well at all concentrations of Fos tested, with significant growth in all wells at or near the levels of no-drug control (Figure 3C). Conversely, the MIC of compound 1 for Fos^R#2^ was 100 μg/ml or 203 μM (IC_50_ of compound 1 for Fos^R#2^ is 24.38 μg/ml, 95% CI = 18.75–31.70 μg/ml; Figure 3D). The MIC was identical between wild-type and Fos^R#2^ and the IC_50_s were very close. The IC_50_ curves can almost be superimposed.

### Is DXR mutated in Fos^R^ bacteria?

Fosmidomycin-resistant bacteria retained sensitivity to compound 1. Since compound 1 is a lipophilic prodrug it will get metabolized to FR900098 inside the bacteria, which will then target bacterial DXR. This suggests that spontaneous resistance is not due to mutation of DXR, since Fos^R^ bacteria are inhibited by compound 1. No transposon insertion mutants are available for DXR as this enzyme is thought to be essential to *Francisella* growth, so the role of DXR could not be directly tested in this study. We thus hypothesized that the mutations that lead to the Fos-resistant phenotype are not mutations in DXR. We then predicted that the GlpT transporter may be mutated, leading to this resistant phenotype. Supporting this, transposon insertion mutants at the *glpT* locus were also found to be resistant to Fos.

### Disk inhibition with *glpT* transposon insertion mutants

The susceptibility of the *glpT* transposon insertion mutants to Fos and FR900098 was also tested in this manner. There was no inhibition of the *glpT* transposon mutant by Fos (Figure 2B), supporting the critical role of *glpT* in Fos transport across the bacterial membranes in order for it to reach the DXR enzyme target inside the bacteria. *glpT* mutants with FR900098 disks demonstrated intermediate inhibition (15 ± 1 mm zone; Figure 2D). This effect of FR900098 on the *glpT* mutant is consistent with our previous studies and published reports suggesting improved bacterial penetration of this compound (McKenney et al., in press). Interestingly, as can be seen in Figure 2D, there were no breakthrough colonies observed in the *glpT* transposon mutant experiments in multiple repeats, perhaps suggesting that the locus was the origination of the instability, leading to the breakthrough colonies observed with the wild-type *F. novicida*, and that other loci are perhaps not as susceptible to mutations leading to breakthrough colonies.

### Antibiotic sensitivity of *glpT* Tn mutants

**Figure 4:**
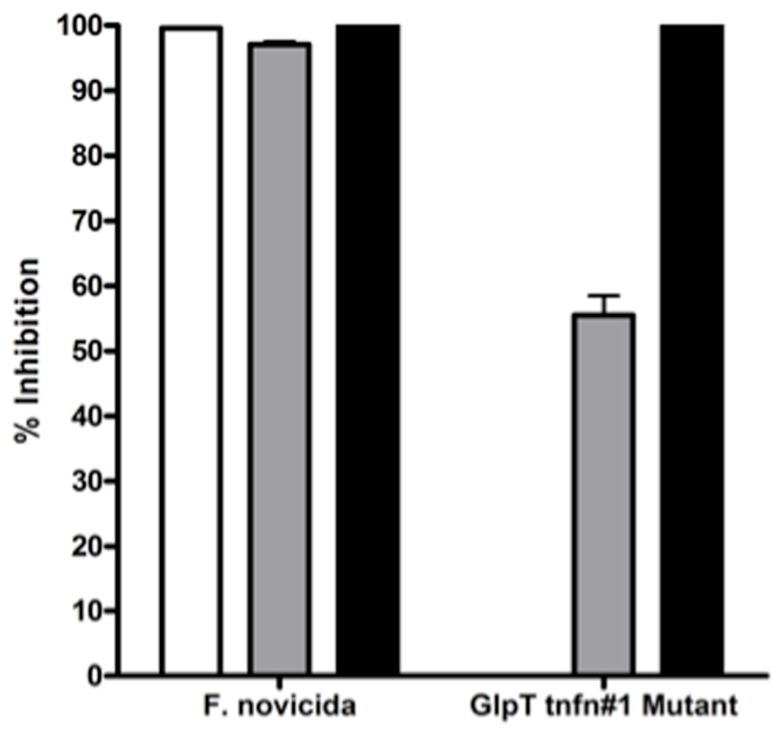
**Sensitivity of wild-type *F. novicida* and *glpT* transposon insertion mutants to fosmidomycin, FR900098, and compound 1**. Sensitivity of *F. novicida* is shown to all three compounds, to fosmidomycin (white bar), FR900098 (gray bar), and the lipophilic prodrug of FR900098, or compound 1 (black bar). Fosmidomycin, FR900098, and compound 1 were tested at concentrations of 200 μg/ml. Resistance of *glpT* transposon insertion mutants to fosmidomycin (white bar) and to a lesser extent FR900098 (gray bar), but not compound 1 (black bar) is shown. No inhibition was seen using fosmidomycin and the transposon mutants. For this experiment, *n* = 3 and error bars represent SEM, and in some cases are too small to be seen.

*glpT* transposon mutants are resistant to Fos and sensitive to compound 1. The *glpT* transposon insertion mutants were tested for their susceptibility to Fos and compound 1 in a liquid broth growth inhibition assay. As expected, the transporter-independent lipophilic prodrug compound 1 was effective at inhibiting growth of the *glpT* transposon insertion mutants (*glpT-1* shown) as well as wild-type *F. novicida*, while Fos at twice the MIC was not (Figure 4). This result supports the hypothesis that mutation in the *glpT* transporter sequence can have a significant detrimental effect on transport of Fos across the bacterial membrane, preventing its interaction with the intended target enzyme, DXR. Intermediate inhibition of the *glpT* mutant by FR900098 was observed in this assay (gray bars) consistent with the disk-inhibition assay results (see Figure 2D), perhaps due to the slightly more lipophilic nature of FR900098 compared to Fos (see Figure 1 for structures), which may allow some bacterial penetration.

### Bioinformatic identification of GlpT

The *glpT* coding region (GlpT) was identified in the *F. tularensis tularensis* SchuS4 (FTT0725c) and *F. novicida* (FTN_0636) genome (accession numbers YP_169738.1 and YP_898283.1) via BLAST search using the *E. coli* K12 homologous sequence (accession number NP_416743.1) as the query. All other *Francisella* sequences also have a highly conserved *glpT* homolog (e.g., FTM_1358, FTW_1513, FTF0725c, FTA_1594, FTH_1462, FTL_1510, Fphi_0200). In addition, bioinformatics examination of *Francisella* genomes revealed no homologous gene for *fsr*, an efflux pump responsible for removal of the Fos from *E. coli* (Messiaen et al., 2011). Also, by our bioinformatic analysis, the published genomes for *F. novicida*, *F. tularensis* Schu S4, and *F. tularensis* LVS also do not contain the *uhpT* transporter (an alternate transporter that could substitute for *glpT*), although the related species *F. philomiragia*, which has an environmental habitat and is not a known pathogen for humans, has a gene that may be *uhpT* (Fphi_0883).

### Sequencing of *glpT* in the Fos^R^ colonies

**Figure 5:**
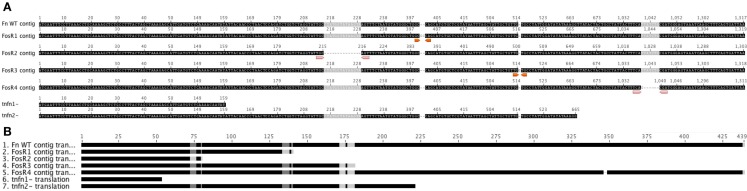
**Sequence of Fos^R^*F. novicidaglpT* vs. WT**. DNA Sequencing of the entire ORF for *glpT* from *F. novicida* reveals nucleotide deletions and additions that result in missense frame shifts and stop codon insertion mutations from isolated colonies grown in the presence of fosmidomycin. *GlpT* transposon mutants are shown for comparison (Gallagher et al., 2007). **(A)** FnFos^R#1^ – GC insertion at nt 401/402 results in frame shift after Try^133^ and stop codon insert at nt 418/420. FnFos^R#2^ – 14 bp deletions at nt 216 results in frame shift at Ala^72^ and stop codon insert at nt 238/240. FnFos^R#3^ – G insertions at nt 517 results in frame shift after Val^171^ and stop codon insert at 541/543. FnFos^R#4^ – 6 bp deletion at nt 1042 results in deletion of Ser^346^ and Leu^347^ from the alpha-helix H9. **(B)** Resulting amino acid alignment from the Fos^R^ mutants vs. WT *F. novicida* U112.

**Figure A2:**
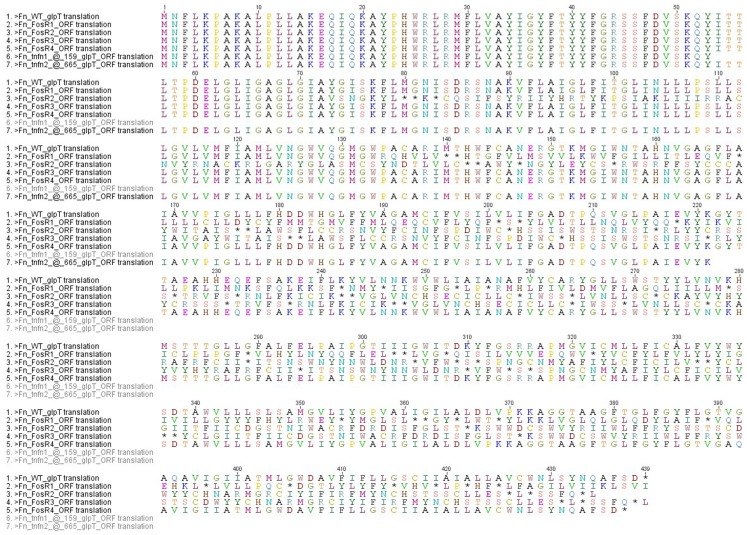
**Full amino acid sequence data for Fos^R^ mutants vs. WT *F. novicida***. Translation of the full length DNA sequences including the positions of amino acid substitution and appearance of early stop codon insertions.

In order to examine the sequence of *glpT* in the Fos^R^ colonies, we isolated the *glpT* region by PCR and sequenced *glpT* from four different spontaneous Fos^R^ colonies. Colonies were picked and grown in liquid culture overnight in TSB-C to create stocks and small aliquots were removed for genomic DNA preparation and sequencing of *glpT*. Comparison of sequences of the four selected Fos^R^ mutants with wild-type sequence data reveals an array of nucleotide deletions and additions from various portions of the *glpT* gene (Figure 5; Figure A1 in Appendix). The 14 nucleotide deletion from mutant FnFos^R#2^ results in a deletion of amino acids in the translated protein as well as the formation of additional stop codons. Similarly, the addition of two nucleotides and one nucleotide in FnFos^R#1^ and FnFos^R#3^, respectively, results in missense mutation by amino acid substitutions and the appearance of premature stop codons resulting in truncated proteins. These abbreviated proteins are likely non-functional. FnFos^R#4^ has a single deletion of six nucleotides at position 1126 of the open reading frame, corresponding to the deletion of a Leu^347^ and Ser^348^ amino acid residues in the protein (Figure 5; Figure A2 in Appendix). When the amino acid sequence of GlpT from the FnFos^R#4^ is compared with *E. coli*, the deleted amino acids occur in a region that is analogous to a peripheral alpha-helix number H9 that spans the lipid membrane (Lemieux et al., 2005) but is not associated with the formation of the pore through which Fos is transported into the cell.

### Rate and frequency of fosmidomycin resistance of *F. novicida*

We determined the rate and frequency of Fos^R^ of *F. novicida*. Using the Drake (1969) formula, the mutation frequency (*f*) and the mutation rate (μ) resulting in *F. novicida* Fos^R^ are approximately 6.3 × 10^−8^ and 0.173, respectively. The *f* is defined as the number of observed colonies in the presence of the inhibitor divided by the total number of cells in culture (*N*_t_). The μ is determined by the number of observed colonies (*m*), the frequency of mutation (*f*), and the *N*_t_ (Table 1). Similarly, the rate determined by the Maximum Likelihood Method is 0.211 (95% CI = 0.159–0.268; Rosche and Foster, 2000).

**Table 1:** **Frequency of mutants resistant to fosmidomycin (122 μM) from the wild-type strain of *F. novicida* U112**.

Total bacteria (*N*_t_)	Resistant colonies	Frequency (*f*)	Rate (μ)
4.81 × 10^10^	270		
5.18 × 10^10^	1500		
**3.66 × 10^10^**	**2310**	**6.3 × 10^−8^**	**0.173**
3.96 × 10^10^	2520		
4.17 × 10^10^	2820		

*First column represents five different aliquots of bacterial culture as described, and the second column represents the number of resistant colonies obtained when plated on 122 μM fosmidomycin. Bold signifies the value selected to determine Rate and Frequency*.

## Discussion

*Francisella* DXR is inhibited by Fos and FR900098. We have previously established that Fos inhibits purified DXR from *F. tularensis* LVS with half maximal activity of 247 nM (Jawaid et al., 2009), comparable to its effect against DXR from *M. tuberculosis* (310 nM; Dhiman et al., 2005). In addition, we previously demonstrated that FR900098 was found to have an IC_50_ of 230 nM against purified *F. tularensis* LVS DXR (McKenney et al., in press). In this study, we have demonstrated the sensitivity of *F. tularensis tularensis* (NIHB38) and *F. novicida* to Fos and FR900098 through the use of Kirby–Bauer disk-inhibition assays.

*Francisella* species are sensitive to Fos through their ability to transport the drug via the glycerol-3-phosphate transporter (GlpT) and expression of the target enzyme DXR. Spontaneous mutants (Fos^R^) of *Francisella* (including Type A) were observed that contained mutations in *glpT*, suggesting a transporter-based mechanism of resistance. Fos has been shown to be effective against *Plasmodium falciparum*, the causative agent of malaria (Jomaa et al., 1999), and some Gram-negative bacteria *in vivo* (Neu and Kamimura, 1981). Spontaneous resistance to Fos has been observed with *E. coli* (Kanimoto and Greenwood, 1987). Known mechanisms of resistance to Fos include overexpression of the target enzymes DXR or IspD (Zhang et al., 2011), overexpression of efflux pumps (*fsr*) to remove the drug from the bacterial cell (Messiaen et al., 2011), and mutation or down-regulation of the GlpT transporter (Fujisaki et al., 1996; Sakamoto et al., 2003; Brown and Parish, 2008; Messiaen et al., 2011).

The proposed mechanism of resistance to Fos in *Burkholderia cepacia* involves both the lack of a functional GlpT transporter and an increased expression of an efflux pump (*fsr*) responsible for removal of the drug from the cell (Messiaen et al., 2011). Bioinformatics examination of *Francisella* genomes revealed no homologous *fsr* efflux pump. Upon bioinformatic analysis, the published genomes for *F. novicida*, *F. tularensis* Schu S4, and *F. tularensis* LVS also do not contain the *uhpT* transporter (an alternate transporter that could substitute for *glpT*), although the related environmental species *F. philomiragia* does appear to have *uhpT*. The reason for this difference is unknown.

Thus, the discovery and development of transporter-independent Fos derivatives could represent a viable strategy to combat zoonotic bacterial infections, or microbes that have been developed as bioweapons. Although *Staphylococcus aureus* and some other organisms lack a functional DXR homolog, bacteria that contain DXR include *B. anthracis* (Ravel et al., 2009) and *Y. pestis* (Pieper et al., 2008). Fos^R^ is perhaps surprising to see in *Francisella* given that there is little evidence that spontaneous resistance to other antimicrobials occurs readily in this organism (Kormilitsyna and Marakusha, 1983).

1-Deoxy-d-xylulose 5-phosphate reductoisomerase is an essential enzyme in organisms that use the MEP pathway, as mutations have been shown to be lethal (Gallagher et al., 2007; Brown and Parish, 2008). For this reason, antibiotics that target DXR have a great potential as broad-spectrum therapeutics. While mutations could occur in the DXR active site that would alter drug activity, complete knockouts of this enzyme are unlikely due to it being an essential enzyme. Interestingly, there were no breakthrough colonies observed in the disk assays with the *glpT* transposon mutants, suggesting that this locus may be responsible for the relatively high number of breakthrough colonies observed with the wild-type *F. novicida*. This finding suggests that mutations of DXR do not readily occur, even under selection with Fos, supporting the use of DXR as a validated target for future antimicrobial drug development. This also supports the study of lipophilic prodrugs such as compound 1 as potential broad-spectrum antimicrobial compounds (Obiol-Pardo et al., 2011; Uh et al., 2011; Ponaire et al., 2012).

It is shown here that mutations in the *glpT* gene can render *F. novicida* resistant to high levels of Fos (both Fos^R^ and *glpT* transposon insertion mutants). This is supported by the data showing that the transposon insertion mutants of *glpT* are also resistant to Fos. By using the lipophilic prodrug of FR900098 (compound 1), it is evident that both the spontaneous *glpT* transposon insertion mutants and Fos^R^ mutants are still susceptible to compound 1, consistent with its transporter-independent mechanism of entry into the bacteria. This result also suggests that the spontaneous resistance is not due to mutations in DXR, the intracellular bacterial target of Fos, FR900098, and compound 1. It has recently been shown that Fos also targets the enzyme following DXR in the MEP pathway, 2-C-methylerythritol-4-phoshpate cytidyltransferase (or IspD) in *E. coli* and *P. falciparum* (Zhang et al., 2011). This is a promising discovery, as there is a lower frequency of resistance to antibiotics that have multiple targets (Silver, 2011).

Developing novel therapeutics based on lipophilic derivatives of phosphonic acids such as Fos and FR900098 represents a new approach to antimicrobial development with the potential for broad-spectrum activity against various zoonotic agents (Ortmann et al., 2003; Jawaid et al., 2009; Wiemer et al., 2010; Davey et al., 2011). Compound 1 was recently shown to be effective against a wide range of bacteria (Obiol-Pardo et al., 2011; Uh et al., 2011; Ponaire et al., 2012). Such compounds may also be effective against many biothreat agents (Jawaid et al., 2009). The conservation of DXR and *glpT* in the genus *Francisella* suggests that transporter-dependence of any Fos-derived compounds should be considered early in the development of these compounds as potential antibiotics. Furthermore, the development of such compounds to be transporter-independent would be most desirable. Importantly, these compounds would retain their effectiveness against strains that may be engineered for other mechanisms of antimicrobial resistance due to the fundamental requirement for DXR in most bacterial biothreat agents.

## Conflict of Interest Statement

The authors declare that the research was conducted in the absence of any commercial or financial relationships that could be construed as a potential conflict of interest.
